# Antioxidant Systems and Quality in Sweet Cherries Are Improved by Preharvest GABA Treatments Leading to Delay Postharvest Senescence

**DOI:** 10.3390/ijms25010260

**Published:** 2023-12-23

**Authors:** Alberto Carrión-Antolí, Fátima Badiche-El Hilali, José M. Lorente-Mento, Huertas M. Díaz-Mula, María Serrano, Daniel Valero

**Affiliations:** 1 Department of Food Technology, EPSO-CIAGRO, University Miguel Hernández, Ctra. Beniel, km 3.2, 03312 Orihuela, Alicante, Spain; alberto.antoli@umh.es (A.C.-A.); fbadiche@umh.es (F.B.-E.H.); 2 Department of Applied Biology, EPSO-CIAGRO, University Miguel Hernández, Ctra. Beniel, km 3.2, 03312 Orihuela, Alicante, Spain; jlorente@umh.es (J.M.L.-M.); h.diaz@umh.es (H.M.D.-M.)

**Keywords:** anthocyanins, ascorbate peroxidase, catalase, firmness, peroxidase, phenolics, *Prunus avium* L., storage

## Abstract

γ-Aminobutyric acid (GABA) plays important roles in plant development, including the maintenance of fruit quality when applied as postharvest treatment. However, little information is available about the effects of preharvest GABA treatments. Thus, GABA (10, 50 and 100 mM) was applied as foliar spray at key points of fruit development in three sweet cherry cultivars and over two years. The results show that quality parameters, such as total soluble solid content, titratable acidity and firmness were higher in the fruit from GABA-treated trees than in the controls, either at harvest or during four weeks of cold storage. In addition, the total phenolic and total and individual anthocyanin concentrations were also enhanced by GABA treatments and the fruit color was improved. The activities of the antioxidant enzymes catalase, ascorbate peroxidase and peroxidase were also enhanced by the GABA treatments. The most effective concentration was 50 mM, which led to extending the storage period of sweet cherries with high quality traits to up to four weeks, while for the controls this was two weeks. Thus, GABA treatment had a clear effect on delaying the postharvest ripening and senescence processes in sweet cherries, with an additional effect on enhancing the content of bioactive compounds, such as phenolics and anthocyanins, with antioxidant properties and health benefits.

## 1. Introduction

Sweet cherry (*Prunus avium* L.) fruit have high-quality properties, namely juiciness, texture, color, taste and flavor, making them highly appreciated by consumers around the world [1,2,3,4]. In addition, they have bioactive compounds, such as phenolic acids, anthocyanins, flavonoids and ascorbic acid, which have antioxidant properties and are the major responsible actors for the beneficial effects of sweet cherry consumption to human health, which include lower risk of suffering from degenerative illnesses, such as diabetes, cardiovascular, inflammatory and several kinds of cancer, among others [5,6,7]. However, the postharvest ripening and senescence processes evolve rapidly in sweet cherry fruit and their quality deteriorates in a short period of time. The most usual practice to maintain fruit quality is storage at cold temperature as soon as possible after harvest, but even in this case, the shelf life of cherries is no longer than 2–3 weeks, depending on cultivar and other preharvest factors [8,9]. In this sense, the combination of cold storage with other postharvest technologies, such as edible coatings based on alginate [10], *Aloe vera* gel, alone or combined with aromatic plant extracts [11], and chitosan [12] or nano-silica coating combined with pressurized Ar [13], 1-methylcyclopropene [14,15] or salicylates and oxalic acid [16] treatments, led to the maintenance of the sweet cherry fruits’ quality properties and the extension of their shelf life. On the other hand, preharvest treatments with salicylic acid, acetyl salicylic acid or methyl salicylate [17], as well as with oxalic acid [2], gibberellic acid [18], melatonin [19,20] or methyl jasmonate [21] proved to have important effects on increasing fruit quality traits (size, color, firmness and sugar content) at harvest and on their maintenance at higher levels, as compared with fruit from control trees during storage.

γ-Aminobutyric acid (GABA) is a four-carbon non-protein amino acid which plays important roles in plants and animals. In humans, GABA has many health-related effects, acting against inflammatory, diabetic, hypertensive and cancer illnesses [22]. In plants, GABA has been reported to regulate many plant physiological processes; the first ones discovered being the plant resistance induction to abiotic and biotic stresses [23,24,25]. Glutamate is the precursor for GABA synthesis by action of glutamate decarboxylase, and GABA could be metabolized through the GABA shunt pathway, rendering α-ketoglutarate and succinate in two consecutive reactions. In addition, the GABA shunt plays important roles in reducing reactive oxygen species (ROS) and acting as a signaling molecule regulating several stress-related mechanisms [25,26]. More recently, beneficial effects of postharvest fruit GABA treatments on maintaining quality properties and reducing chilling injury damage have been reported in a wide range of fruit species, such as table grape [27], cornelian [28], peaches [29], mango [30], loquat [31] and kiwifruit [32], among others.

However, the literature regarding the effects of GABA application as preharvest treatment in fruit quality attributes is scarce. Foliar spray with GABA solutions of pomegranate trees led to increased crop yield and fruit quality properties at harvest, which were also maintained at higher levels during storage as compared to fruit from control trees. In ‘Fino-95’ lemon, the GABA treatment of the trees increased crop yield (with ca. 15% kg per tree with respect to controls), without affecting fruit firmness, total soluble solids or titratable acidity [33]. Finally, GABA foliar spray application to apple trees, 1 or 2 weeks before harvest, decreased soft scald symptoms after cold storage, although no effects were observed in other fruit quality parameters [34].

Specifically, in sweet cherry fruit, no previous reports are available about GABA treatments either applied as post- or as preharvest treatments, although Wang et al. [35,36] assayed the effects of postharvest treatments with β-aminobutyric acid (BABA), a GABA isomer. In these papers, it was reported that BABA dipping treatment for 10 min reduced weight loss and softening and maintained high levels of sugars, organic acids, phenolics and antioxidant enzyme activities during storage at 20 °C, leading to maintaining overall sweet cherry fruit quality.

According to the previous literature, it was hypothesized that preharvest GABA treatments could have beneficial effects on sweet cherry fruit quality traits either at harvest or during storage, which was the main goal of the present experiments. In addition, it was important to know whether these effects could be dependent on cultivar or growing season. For this purpose, different GABA concentrations were assayed in three sweet cherry cultivars and for two years, in order to obtain more broad conclusions regarding the effects of GABA.

## 2. Results

### 2.1. Sweet Cherry Quality Parameters during Storage

**Figure 1:**
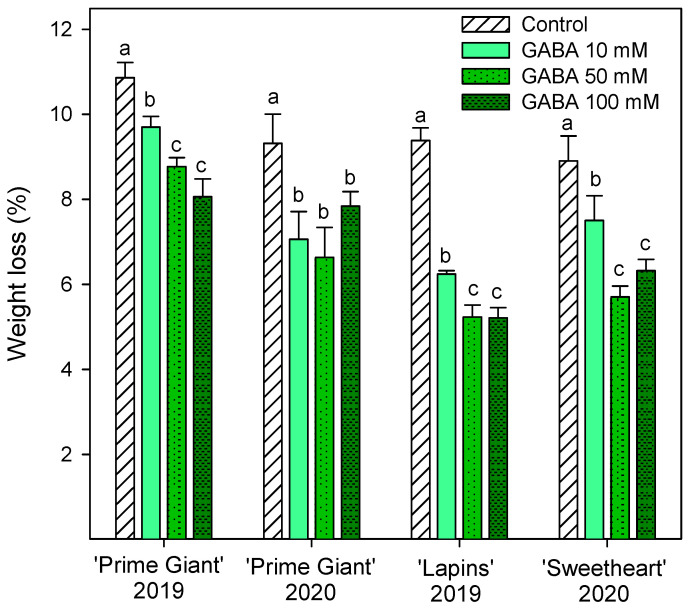
Weight loss of sweet cherries from control and γ-aminobutyric acid (GABA)-treated trees after 28 days of storage at 2 °C. Data are the mean ± SE of three replicates. Different letters show significant differences (*p* < 0.05) among treatments for each cultivar and growing cycle.

**Figure 2:**
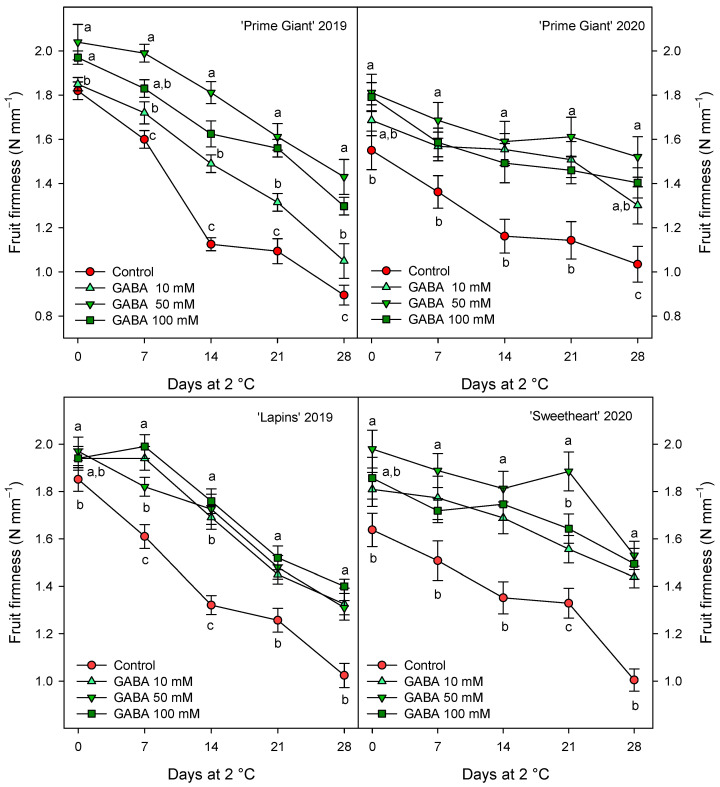
Fruit firmness of sweet cherries from control and γ-aminobutyric acid (GABA)-treated trees during 28 days of storage at 2 °C. Data are the mean ± SE of three replicates. Different letters show significant differences (at *p* < 0.05) among treatments for each sampling date, for each cultivar and growing cycle.

Weight loss increased during storage in all cherry cultivars, reaching final values in control fruits of 9–10% after 28 days of storage, depending on the cultivar and growing cycle. However, weight losses were significantly lower (*p* < 0.05) in fruit from GABA-treated trees, the lowest weight losses being found for 50 and 100 mM concentrations (Figure 1). With respect to fruit firmness, significant effects of GABA treatments were also observed, since firmness values were higher in fruit from GABA-treated trees than in controls, either at harvest or during the whole storage time. In general, the highest effects (*p* < 0.05) on diminishing firmness losses during storage were observed for the 50 mM GABA treatment, although for “Lapins” in the 2019 experiment, no significant differences were observed among the GABA-applied doses (Figure 2). In addition, it is worth noting that the fruit firmness of “Lapins” from the 50 mM GABA treatment was 17% higher than for the controls, while for the remaining cultivars’ firmness values were 30–35% higher than in the controls during the whole storage periods. Thus, the effect of preharvest GABA treatments on maintaining this important quality trait was lower for the “Lapins” cultivar.

Total soluble solids (TSSs) and titratable acidity (TA) were also significantly affected (*p* < 0.05) by GABA treatments, either at harvest or during storage (Table 1). Thus, TSS and TA values at harvest were increased as a consequence of the GABA treatments, the effect being dose-dependent for TSSs in “Prime Giant” and “Sweetheart”, while for ‘Lapins’ similar values were obtained with all the GABA doses assayed as well as for TA for all cultivars and growing cycles (Table 1). During storage, a significant increase in TSSs occurred, although at the end of the storage time the TSS content was still higher in the fruit from the GABA-treated trees than in the controls (Table 1). On the contrary, TA significantly decreased during storage whether in the controls or in the treated fruit. However, the TA decreasing rate was delayed by the GABA treatments since, at the end of storage, the TA values were significantly higher in fruit from treated trees than in the controls (Table 1) for all cultivars and years.

### 2.2. Phenolic and Anthocyanin Concentrations during Storage

**Figure 3:**
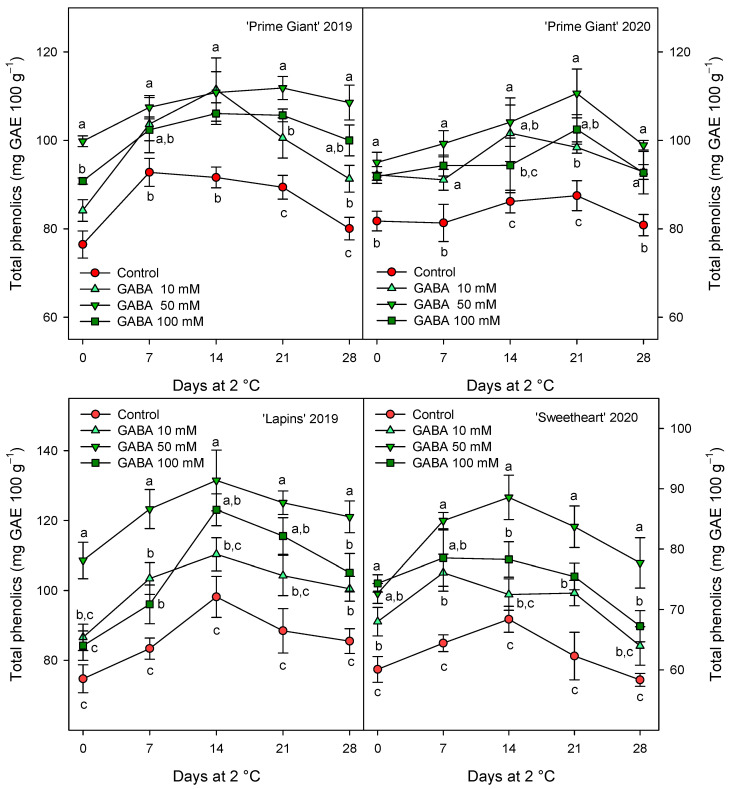
Total phenolic content (mg gallic acid equivalent (GAE) 100 g^−1^) in sweet cherries from control and γ-aminobutyric acid (GABA)-treated trees during 28 days of storage at 2 °C. Data are the mean ± SE of three replicates. Different letters show significant differences (at *p* < 0.05) among treatments for each sampling date, for each cultivar and growing cycle.

**Figure 4:**
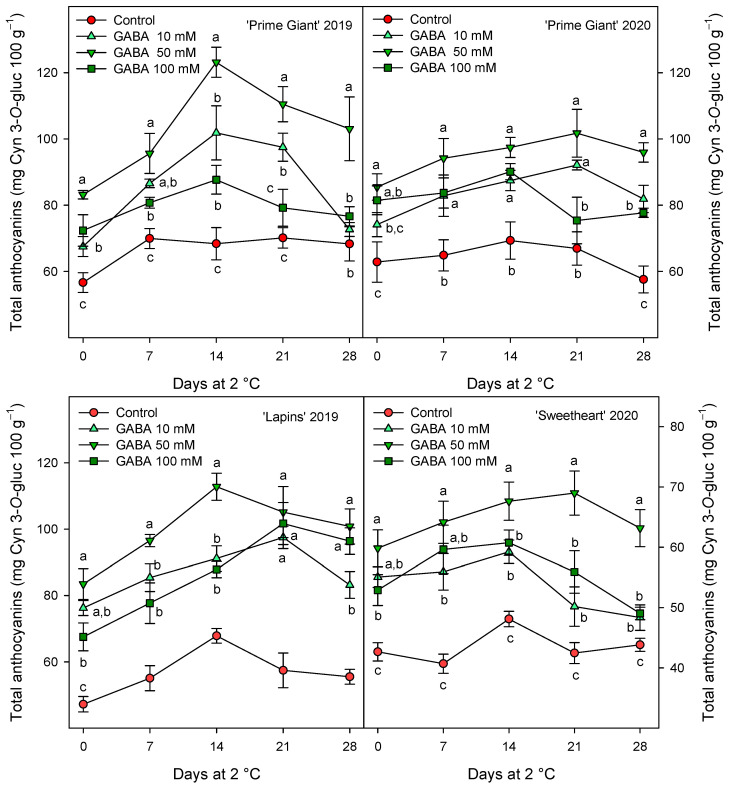
Total anthocyanin content (mg cyanidin 3-*O*-gluciside equivalent (Cyn 3-*O*-gluc) 100 g^−1^) in sweet cherries from control and γ-aminobutyric acid (GABA)-treated trees during 28 days of storage at 2 °C. Data are the mean ± SE of three replicates. Different letters show significant differences (at *p* < 0.05) among treatments for each sampling date, for each cultivar and growing cycle.

**Figure 5:**
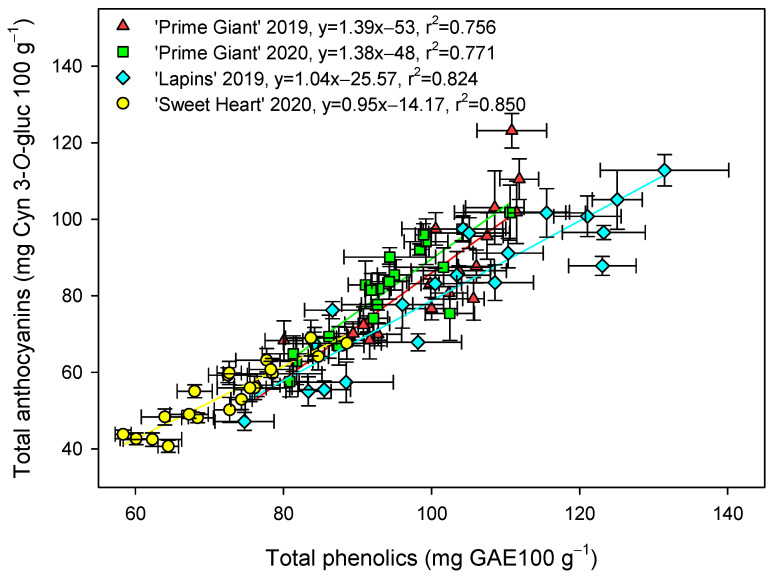
Correlation between total phenolic (mg gallic acid equivalent (GAE) 100 g^−1^) and anthocyanin (mg cyanidin 3-*O*-glucoside equivalent (Cyn 3-*O*-gluc) 100 g^−1^) concentrations for each cultivar and year taking into account data from control and treated fruit and sampling dates. Data are the mean ± SE of three replicates.

The total phenolic concentration was significantly (*p* < 0.05) enhanced by preharvest GABA treatments, and in general, the highest effect was found for the 50 mM dose from day 0 to the end of storage for all cultivars and years (Figure 3). Nevertheless, this effect was more dependent on cultivar than on growing cycle, since increases ca. 25% were observed for “Prime Giant” in 2019 and 2020, and ca. 30 and 40% for “Sweetheart” in 2020 and “Lapins” in 2019, respectively. Similarly, significantly higher (*p* < 0.05) concentrations of total anthocyanins were observed in sweet cherries from GABA-treated trees as compared with those from the controls, during the whole storage period (Figure 4). The highest increases in this parameter were also found for the 50 mM dose, which were of H75 % for “Lapins” in 2019 and H50% for “Sweetheart” in 2020 and for “Prime Giant” in both years. In addition, a similar trend was found for total phenolic and anthocyanin concentrations during storage, with increases from day 0 to day 14–21 and decreases thereafter, independently of the treatments, cultivars or years (Figure 3 and Figure 4). In fact, high correlations (r^2^ = 0.76 − 0.85) were observed between phenolic and anthocyanin concentrations for all cultivars and years when data from all sampling dates were considered (Figure 5).

**Figure 6:**
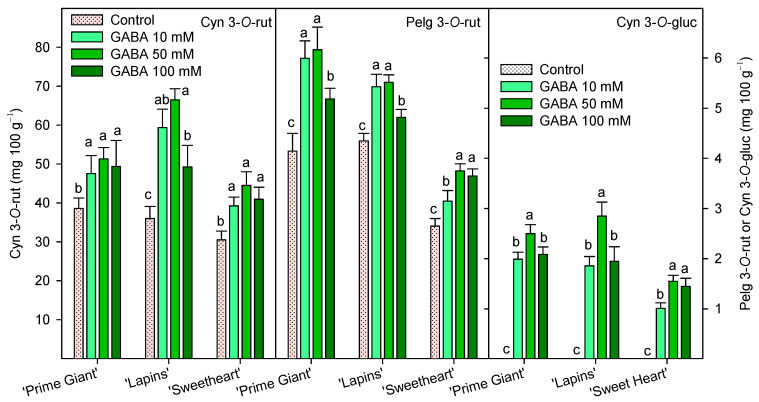
Individual anthocyanin concentration, cyanidin 3-*O*-rutinoside (Cyn 3-*O*-rut, left axis), pelargonidin 3-*O*-rutinoside (Pelg 3-*O*-rut, right axis) and cyanidin 3-*O*-glucoside (Cyn 3-*O*-gluc, right axis), at harvest (day 0, 2019 for “Prime Giant” and “Lapins” and 2020 for “Sweetheart”) in sweet cherries from control and γ-aminobutyric acid (GABA)-treated trees. Data are the mean ± SE of three replicates. Different letters show significant differences at *p* < 0.05 for each cultivar and growing cycle.

Individual anthocyanin concentration was measured at harvest (day 0, 2019 for “Prime Giant” and “Lapins” and 2020 for “Sweetheart”) and the results show that cyanidin 3-*O*-rutinoside (Cyn 3-*O*-rut) was the major one, followed by pelargonidin 3-*O*-rutinoside (Pelg 3-*O*-rut) and cyanidin 3-*O*-glucoside (Cyn 3-*O*-gluc). These three individual anthocyanins were significantly (*p* < 0.05) enhanced by GABA treatments in all cultivars, and in general, the highest increases were observed for the 50 mM GABA dose (Figure 6).

### 2.3. Antioxidant Enzymes

**Figure 7:**
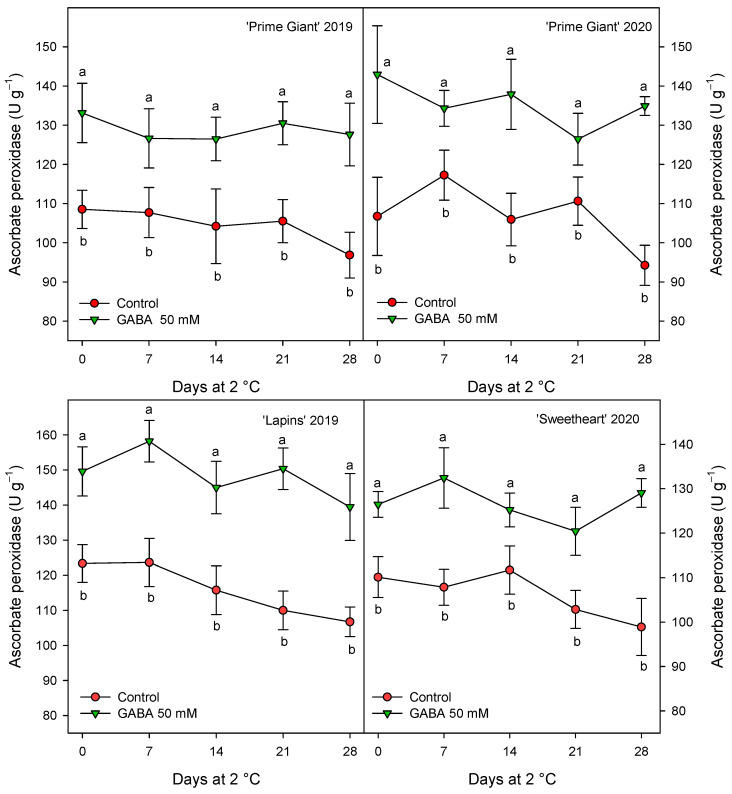
Ascorbate peroxidase activity in sweet cherries from control and γ-aminobutyric acid (GABA)-treated trees during 28 days of storage at 2 °C. Data are the mean ± SE of three replicates. Different letters show significant differences (at *p* < 0.05) between treatments for each sampling date, for each cultivar and growing cycle.

**Figure 8:**
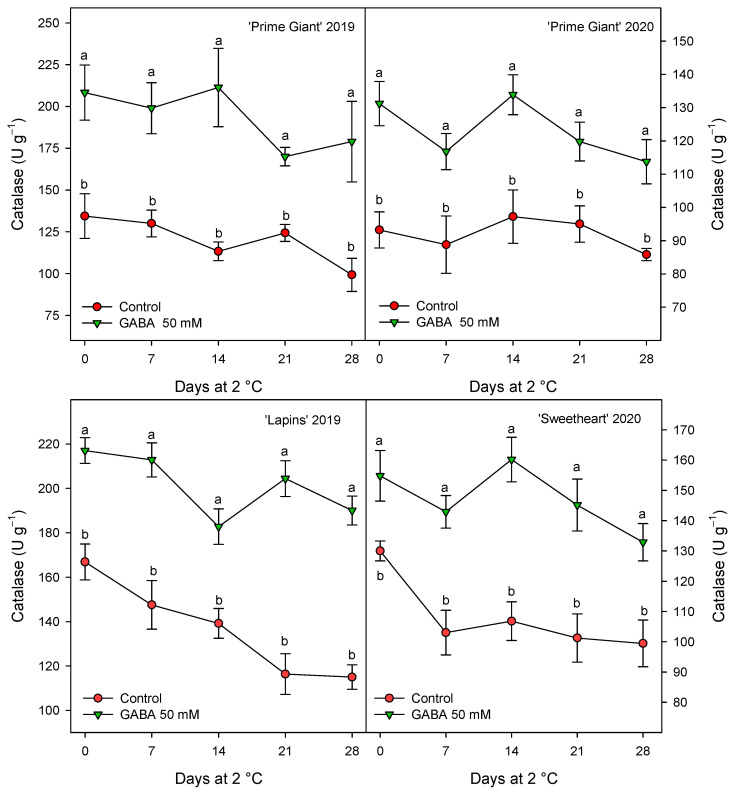
Catalase activity in sweet cherries from control and γ-aminobutyric acid (GABA)-treated trees during 28 days of storage at 2 °C. Data are the mean ± SE of three replicates. Different letters show significant differences (at *p* < 0.05) between treatments for each sampling date, for each cultivar and growing cycle.

**Figure 9:**
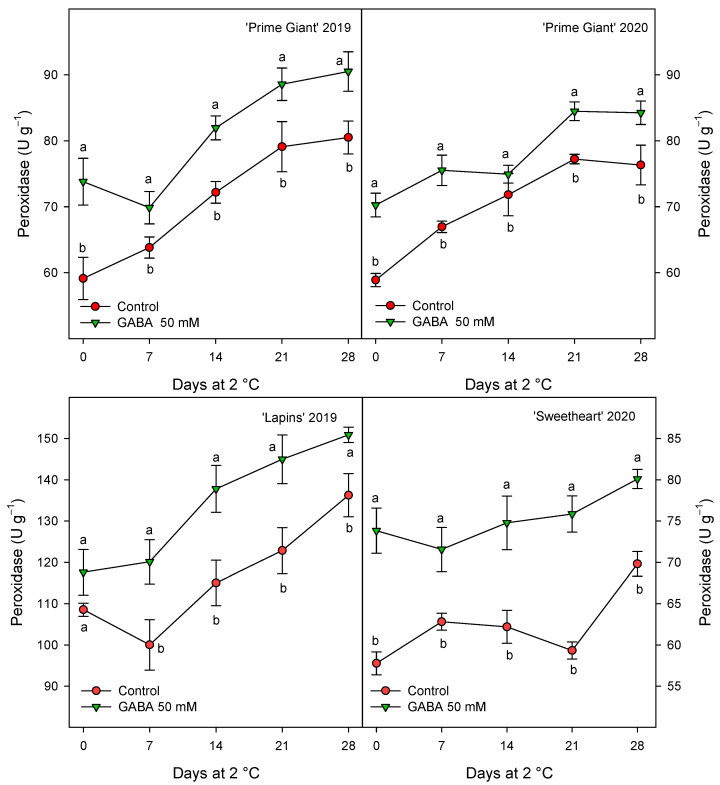
Peroxidase activity in sweet cherries from control and γ-aminobutyric acid (GABA)-treated trees during 28 days of storage at 2 °C. Data are the mean ± SE of three replicates. Different letters show significant differences (at *p* < 0.05) between treatments for each sampling date, for each cultivar and growing cycle.

Antioxidant enzymes, APX, CAT and POD were measured in sweet cherries from control and 50 mM GABA-treated trees since, generally, this dose was the more effective for maintaining higher values of quality parameters and antioxidant compounds during storage. The results show significantly higher values (*p* < 0.05) in cherries from treated trees than in controls during the whole storage period (Figure 7, Figure 8 and Figure 9). For APX activity, a decrease trend in the control fruits was observed after 14–21 days of storage while, for fruit from GABA-treated trees, this activity remained at similar levels than at harvest during the whole storage time (Figure 7). However, it is worth noting that the effects of GABA treatment were similar for all cultivars and years, with increases ranging from 20 to 28% when data for all sampling dates were taken into account.

CAT activity was also found at higher levels in fruits from GABA-treated trees as compared with controls, although for this activity the highest increases, ca. 60%, were observed for “Prime Giant” in 2019, followed by “Lapins” in 2019, ca. 50%, while for “Prime Giant” and “Sweetheart” in 2020, the increases were ca. 35% (Figure 8). Finally, POD activity was also significantly increased by GABA treatment, although these increases were lower than those for APX and CAT activities, since they were of 15–20% with respect to the controls, independently of the cultivar or growing cycle (Figure 9).

## 3. Discussion

The main quality attributes of sweet cherry fruit are visual appearance, such as absence of defects, size and color; stem freshness and length; organoleptic properties including juiciness, firmness, sweetness, sourness, taste, aroma and flavor; sweetness and sourness which affect consumer purchase intentions, and vary depending on the cultivars [1,3,8]. However, these quality traits decrease rapidly after harvest, mainly due to stem browning and fruit weight, firmness and acidity losses, leading to decreased fruit juiciness and freshness, so the fruits lose their organoleptic properties and taste over-ripened [9,12,17,36,37]. The present results show that preharvest GABA treatments enhanced red color, fruit firmness and TSS and TA contents at harvest, leading to fruit with higher organoleptic properties. According to the previous reports commented above, increases in fruit weight loss (Figure 1), due to dehydration by transpiration process, and in TSSs (Table 1), as well as decreases in fruit firmness (Figure 2) and TA (Figure 3) were observed in sweet cherries during cold storage. However, these changes were significantly delayed in fruit from GABA-treated trees as compared with the controls in the “Prime Giant” and “Lapins” cultivars in the 2019 experiment, and these effects were confirmed for the “Prime Giant” cultivar and for the “Sweetheart” cultivar in the 2020 experiment and, in general, the highest effects were observed for the 50 mM concentration. Taking into account the results of all these quality parameters, control cherries could be stored at cold temperatures for two weeks with optimal properties for consumption, while this period was extended to up to four weeks for cherries from the 50 mM GABA-treated trees. Postharvest GABA dipping treatments have been proven to successfully maintain fruit quality properties in cornelian cherry [28], loquat [31], peach [29] and tomato [38], with additional effects on reducing chilling injury symptoms. Moreover, other postharvest treatments with effects on maintaining fruit quality, such as sodium nitroprusside (SNP) in peach [39], calcium in apple [40] or melatonin [41] and methyl jasmonate [42] in tomato, increased GABA content and the GABA-shunt pathway, proving the effects of GABA on delaying the postharvest ripening and senescence processes, which has been attributed to an enhanced mitochondrial energy status [29]. It is important to note that consumers have concerns about post-harvest fruit treatments, which have also more legal restrictions than preharvest ones; meanwhile, preharvest treatments with GABA, which is a natural amino acid, are considered to be safe and to have beneficial properties for human health [43], so this could be a suitable and environmentally friendly approach to increase sweet cherry quality and their storage time for longer periods.

The content of phenolic compounds and especially anthocyanins in sweet cherry fruit have attracted increasing interest in recent years due to their antioxidant properties responsible for their positive impact on human health, namely by reducing the risk of suffering from degenerative diseases [7,8,44,45,46]. In the present results, a significant increase in total phenolic and anthocyanin content was observed as a consequence of GABA treatment, either at harvest or during the whole storage period (Figure 3 and Figure 4). In general, total phenolic and anthocyanin concentrations increased from day 0 to day 7–14 in control fruit and decreased thereafter, these changes being related to the evolution in the postharvest ripening process of sweet cherry [9,16,21], which was delayed in the fruit from GABA-treated trees. In addition, a higher content in individual anthocyanin concentration was found in treated cherries at harvest as compared with controls (Figure 6). The major anthocyanin in “Prime Giant”, “Lapins” and “Sweetheart” was cyn 3-*O*-rut in the three cultivars, in agreement with previous reports for other cultivars [2,4,9,46,47,48,49]. No previous studies are available in the literature regarding the effects of preharvest GABA treatments on the biosynthesis of phenolic compounds during fruit on-tree development for comparative purposes, although some reports have been published for postharvest treatments. For instance, GABA dipping treatment increased phenolics and flavonoids in carambola [50,51], cornelian cherry fruits [28] and tomato [52], as well as in fresh pistachio fruit [53], although in the last fruit species higher effects were observed when GABA was combined with carboxymethyl cellulose coating and CaO. These effects, in aonla and carambola fruits, have been attributed to a higher activity of phenylalanine ammonia lyase (PAL) and reduced activity of polyphenol oxidase (PPO), leading to increased total phenols accumulation [50,54]. However, preharvest GABA treatments seem to be more effective in increasing the content in these bioactive compounds, as has been observed in the present experiments and in previous ones with pomegranates [55]. Thus, GABA treatment may lead to increases in the health benefits of sweet cherries, since phenolics and especially anthocyanins exhibit protective roles against heart, vision and neurological diseases, among others [44,45,46,56]. These effects are attributed to their widely recognized antioxidant, anti-inflammatory and antiapoptotic properties, which also depend on the anthocyanins which significantly depend on the human being’s gut microbiota activities [57].

Oxygen free radicals (ROS), mainly H_2_O_2_, O_2_^−•^ and OH^−•^, accumulate during fruit ripening and senescence, leading to protein and DNA damage membrane lipid peroxidation and senescence process acceleration [58]. Vegetable cells have antioxidant systems able to scavenge these ROS and repair de oxidative damage, including antioxidant compounds (namely ascorbic acid, phenolic compounds, tocopherols and carotenoids) and antioxidant enzymes, such as POD, CAT, APX and superoxide dismutase (SOD), among others [58,59]. The activity of the antioxidant enzymes APX, CAT and POD was found to be higher in cherries from the 50 mM GABA-treated fruit than in the controls for the three cherry cultivars at harvest and for all sampling dates during storage (Figure 7, Figure 8 and Figure 9). Thus, the higher antioxidant systems, enzymatic and non-enzymatic ones, found as a consequence of preharvest GABA treatments, could account for delaying post-harvest ripening and senescence processes and being responsible for the maintenance of fruit quality traits. In fact, the accumulation of ROS, such as H_2_O_2_, O_2_^−•^ and OH^−•^, among others, is a general event occurring during fruit ripening and senescence processes [58,59]. Then, enhancing the fruit cell antioxidant system, both enzymatic and non-enzymatic ones would account for the observed delayed senescence and quality traits maintenance. Accordingly, the antioxidant enzymes SOD, CAT, POD, APX and glutathione reductase were increased in blueberry and carambola fruits by postharvest GABA dipping treatment, leading to delay in the senescence process [50,60]. In addition, different pre- and postharvest treatments with effects on delaying quality properties losses in sweet cherries also increased these antioxidant systems. For instance, vacuum cooling [61], chitosan coating [57,62] or the combination of chitosan with Argon [13] enhanced antioxidant enzyme activities and antioxidant compounds, resulting in extending the sweet cherry shelf life [13], as well as preharvest treatments with salicylates [17], oxalic acid [2] or melatonin [20].

## 4. Materials and Methods

### 4.1. Plant Material and GABA Treatments

Field experiments were performed in a commercial farm, located at Jumilla (Murcia, Spain, coordinates UTMX: 463.700 and UTMY: 4.268.900) by using three replicates of three trees for each cultivar and GABA treatment. In 2019, the assays were made with “Prime Giant” and “Lapins” cultivars, which were 7 years old, and for 2020, the experiment was repeated with “Prime Giant” and a new cultivar, “Sweetheart” (which was 5 years old), was added. Mean annual temperatures in the field during the trials were 15.24 and 15.30 °C for 2019 and 2020, respectively, the accumulated rainfalls were 357 and 352 mm for 2019 and 2020, respectively, and the relative humidity mean values were 60.5 and 65.1%, respectively. All cultivars were grafted onto SL-64 rootstock and were grown under normal agronomic conditions for both years, applying 60:30:100 kg ha^−1^ of N:P:K fertilizers and 5250 m^3^ ha^−1^ of water along the growing cycle and performing an open-center pruning. GABA treatments were performed by applying 3 L of 10, 50 or 100 mM GABA freshly prepared solutions, containing 0.1% Tween 20 as surfactant, as foliar spray with a hand spray machine in order to wet the whole tree canopy. Control trees were treated with tap water containing 0.1% Tween 20. Control and treated trees were separated by other rows of trees to avoid treatment drift. Sweet cherry fruit were harvested at commercial ripening stage, according to characteristic fruit size, color and total soluble solids content of each cultivar, and a sample of 3 kg (1 kg of each tree) was taken for each treatment, replicated and transported to the laboratory (at 15 °C and 70% RH) in 2 h for storage experiments. Once at the laboratory, five lots of 20 fruits, homogeneous in size and color, were selected for each replicate, weighted and stored at 2 °C and 90% RH for 0, 7, 14, 21 and 28 days.

### 4.2. Fruit Quality Parameter Measures

Fruit weight was measured at day 0 and after each storage period, by using a digital balance (KERN 440-35N, Balingen, Germany), and weight loss was expressed as percentage with respect to weight at harvest. Fruit firmness was determined in each individual fruit by using a Texture Analyzer (TX-XT2i model, Stable Mycrosys-tems, Godalming, UK) as previously reported [20], and the results are expressed as N mm^−1^. Then, sweet cherry fruit were cut into small pieces to obtain a homogeneous sample for the 20 fruits of each replicate. One portion of each sample was used for total soluble solids (TSSs) and titratable acidity (TA) measures (immediately after cutting) and another one was frozen and ground under liquid N_2_ and stored at −20 °C until phenolics, anthocyanins and antioxidant enzyme activities were measured. TSSs and TA were measured (in duplicate) in the juice obtained from 50 g of fruit sample, through squeezing and filtration with a double cotton fabric, by a hand refractometer (Atago PR-101, Atago Co. Ltd., Tokyo, Japan) and titration with NaOH 0.1 N until pH 8.1 with the 785 DMP Titrino automatic titration system (Metrohm, Herisau, Switzerland), respectively. TSSs were expressed as °Brix and TA as g of malic acid equivalent to 100 g^−1^.

### 4.3. Measures of Total Phenolic Compounds and Total and Individual Anthocyanins

Phenolics were extracted and quantified according to Carrión-Antolí et al. [23]. Briefly, fruit samples (5 g) were homogenized with 10 mL of water:methanol (2:8, *v*:*v*) plus 2 mM NaF (to avoid phenolic degradation by suppressing the activity of polyphenol oxidase) in an Ultraturrax homogenizer (T18-basic model, IKA, Berlin, Germany). The supernatant was used to quantify total phenolic content (in duplicate in each extract) by addition of the Folin-Ciocalteu reagent as described by Díaz-Mula et al. [1]. The results (mean ± SE) are expressed in gallic acid equivalent, mg 100 g^−1^ on a fresh weight basis. For anthocyanin extraction, 2 g of sample and 10 mL of methanol/water/HCl (80:19:1) were homogenized and centrifuged as described above and anthocyanins were measured in the supernatant by reading absorbance at 530 nm by using an UNICAM Heliosα spectrophotometer (Artisan-Technology-Group, Champaign, IL, USA). The results (mean ± SE) are expressed as cyanidin 3-*O*-glucoside (cyn 3-*O*-gluc) equivalents, taking into account the coefficient of molar absorption of cyanidin 3-*O*-glucoside, 23,900 L cm^−1^ mol^−1^ and its molecular weight, 449.2 g mol^−1^. Anthocyanin extracts were filtered through a 0.45 µm PVDF filter (Millex-HV13, Millipore, Bedford, MA, USA) and used to quantify individual anthocyanins (in duplicate in each extract) in an HPLC analysis system (Agilent HPLC-1200-Infinity series, Santa Clara, CA, USA), according to Martínez-Esplá et al. [2]. Cyn 3-*O*-rut, pelg 3-*O*-rut and cyn 3-*O*-gluc were used to perform standard calibration curves and each individual anthocyanin was expressed as mg 100 g^−1^.

### 4.4. Determination of Antioxidant Enzyme Activities

The extracts for APX, CAT and POD quantification were obtained by homogenizing 5 g of fruit sample with 10 mL of 50 mM phosphate buffer, pH 7.0, with 1 mM EDTA (ethylen–diaminetetraacetic acid) and 1% PVP (polyvinylpyrrolidone) and centrifuging at 15,000× *g* for 30 min at 4 °C [17]. APX quantification was conducted by reading the absorbance at 290 nm for 1 min in a 3 mL volume reaction containing 50 mM potassium phosphate buffer, pH 7.0, 0.5 mM ascorbic acid, 1 mM H_2_O_2_ and 0.1 mL of crude extract. The results are expressed as U g^−1^ and one unit of enzyme activity (U) was defined as a 0.01 absorbance decrease per min. For measuring CAT activity, 0.1 mL of extract was added to 2.9 mL of phosphate buffer (50 mM, pH 7.0), containing 15 mM H_2_O_2_ and the absorbance decrease at 240 nm from 0 time to after 1 min was measured. CAT was expressed as U g^−1^, one U being a 0.01 absorbance decrease per minute. Finally, the reaction mixture for POD measure contained 2.9 mL of phosphate buffer (50 mM, pH 7.0), 12 mM H_2_O_2_, 14 mM guaiacol and 0.1 mL of enzymatic extract. The absorbance was measured at 470 nm at time 0 and after 1 min, and the increase in absorbance due to guaiacol oxidation was calculated and the activity of POD was expressed as U g^−1^. One U was defined as a 0.01 absorbance increase per min.

### 4.5. Statistical Analysis

Field experiments were conducted in a randomized design by using three replicates (of three trees) for each treatment and cultivar in both experimental years. Fruit samples of each replicate were used for storage experiments and, for all the analyzed parameters, sweet cherry cultivar and year data are the mean ± SE of three replicates (n = 3). The SPSS software version 20 (SPSS-Inc., Chicago, IL, USA) was used to perform an analysis of variance (ANOVA) and Tukey’s test was used for mean comparisons to find significant differences among treatments at *p* < 0.05. In addition, linear regressions were performed between total phenolic and anthocyanin content for each cultivar and each year.

## 5. Conclusions

The overall results lead us to conclude that preharvest GABA treatments, especially at a 50 mM dose, make for increased sweet cherry organoleptic quality at harvest which is maintained during storage at higher levels than in control fruits, due to reduced weight, firmness and acidity losses. In addition, antioxidant compounds are enhanced, leading to improved health benefits for sweet cherry fruit consumption. Finally, the higher activity of antioxidant enzymes, together with the higher content in phenolics and anthocyanins, could contribute to reduce the oxidative stress in fruit and to delay the postharvest ripening and senescence process, and in turn, the storage period with proper quality could be extended.

## Figures and Tables

**Table 1:** TSS (°Brix) and titratable acidity (TA) at harvest and after 28 days of storage at 2 °C of sweet cherry fruit from control and γ-aminobutyric acid (GABA)-treated trees.

Cultivar		TSS (Day 0)	TSS (Day 28)	TA (Day 0)	TA (Day 28)
“Prime Giant” 2019	Control	20.43 ± 0.24 ^aA^	22.73 ± 0.17 ^aB^	1.11 ± 0.02 ^aA^	0.85 ± 0.02 ^aB^
	GABA 10 mM	21.88 ± 0.23 ^bA^	24.55 ± 0.35 ^bB^	1.21 ± 0.02 ^bA^	0.96 ± 0.01 ^bB^
	GABA 50 mM	23.03 ± 0.08 ^cA^	25.17 ± 0.41 ^bcB^	1.23 ± 0.03 ^bA^	0.98 ± 0.02 ^bB^
	GABA 100 mM	23.88 ± 0.18 ^dA^	26.32 ± 0.14 ^cB^	1.17 ± 0.04 ^abA^	0.99 ± 0.01 ^bB^
“Prime Giant” 2020	Control	22.63 ± 0.11 ^aA^	23.42 ± 0.15 ^aB^	1.47 ± 0.03 ^aA^	1.12 ± 0.03 ^aB^
	GABA 10 mM	23.47 ± 0.17 ^bA^	25.73 ± 0.45 ^bB^	1.53 ± 0.03 ^abA^	1.27 ± 0.02 ^bB^
	GABA 50 mM	24.08 ± 0.08 ^cA^	27.03 ± 0.23 ^cB^	1.61 ± 0.02 ^bA^	1.37 ± 0.01 ^cB^
	GABA 100 mM	24.57 ± 0.09 ^dA^	26.57 ± 0.33 ^cB^	1.59 ± 0.01 ^bA^	1.36 ± 0.01 ^cB^
“Lapins” 2019	Control	20.90 ± 0.17 ^aA^	21.83 ± 0.08 ^aB^	1.11 ± 0.03 ^aA^	0.86 ± 0.03 ^aB^
	GABA 10 mM	21.85 ± 0.15 ^bA^	23.10 ± 0.35 ^bB^	1.15 ± 0.03 ^abA^	0.98 ± 0.03 ^bB^
	GABA 50 mM	21.92 ± 0.14 ^bA^	22.95 ± 0.16 ^bB^	1.20 ± 0.02 ^bA^	1.03 ± 0.01 ^bB^
	GABA 100 mM	22.02 ± 0.20 ^bA^	23.32 ± 0.09 ^bB^	1.21 ± 0.03 ^bA^	0.99 ± 0.01 ^bB^
“Sweetheart” 2020	Control	20.03 ± 0.21 ^aA^	21.53 ± 0.17 ^aB^	1.32 ± 0.01 ^aA^	1.27 ± 0.02 ^aB^
	GABA 10 mM	21.48 ± 0.23 ^bA^	22.40 ± 0.09 ^bB^	1.39 ± 0.04 ^abA^	1.28 ± 0.01 ^aB^
	GABA 50 mM	22.08 ± 0.04 ^cA^	24.20 ± 0.14 ^cB^	1.45 ± 0.02 ^bA^	1.40 ± 0.02 ^bB^
	GABA 100 mM	21.78 ± 0.20 ^cA^	25.83 ± 0.17 ^dB^	1.44 ± 0.01 ^bA^	1.38 ± 0.01 ^bB^

Different capital letters within a row show significant differences at *p* < 0.05 from day 0 to day 28 for each parameter and treatment. Different lowercase letters within a column show significant differences at *p* < 0.05 among treatments for each cultivar and growing cycle.

## Data Availability

Data are presented in the article and further inquiries could be directed to the corresponding author.
